# Arterial hypertension and sport. Related aspects to certification for physical activity and contraindications to sports practice in hypertensive child and adolescent.

**DOI:** 10.1186/1824-7288-41-S2-A37

**Published:** 2015-09-30

**Authors:** Ugo Giordano

**Affiliations:** 1 Sports Medicine Unit, Department of Pediatric Cardiology and Cardiac Surgery, Bambino Gesù Children's Hospital, Rome, Italy

## 

The control of blood pressure during exercise is a complex process as it involves increases in stroke volume and heart rate, changes in peripheral vascular resistance and in sympathetic tone; it is related to the type of exercise practiced as its intensity and duration, ratio of lean mass/fat mass, depending on the sex. We distinguish two main types of exercise, aerobic or dynamic and isometric or static.

**Figure 1:**
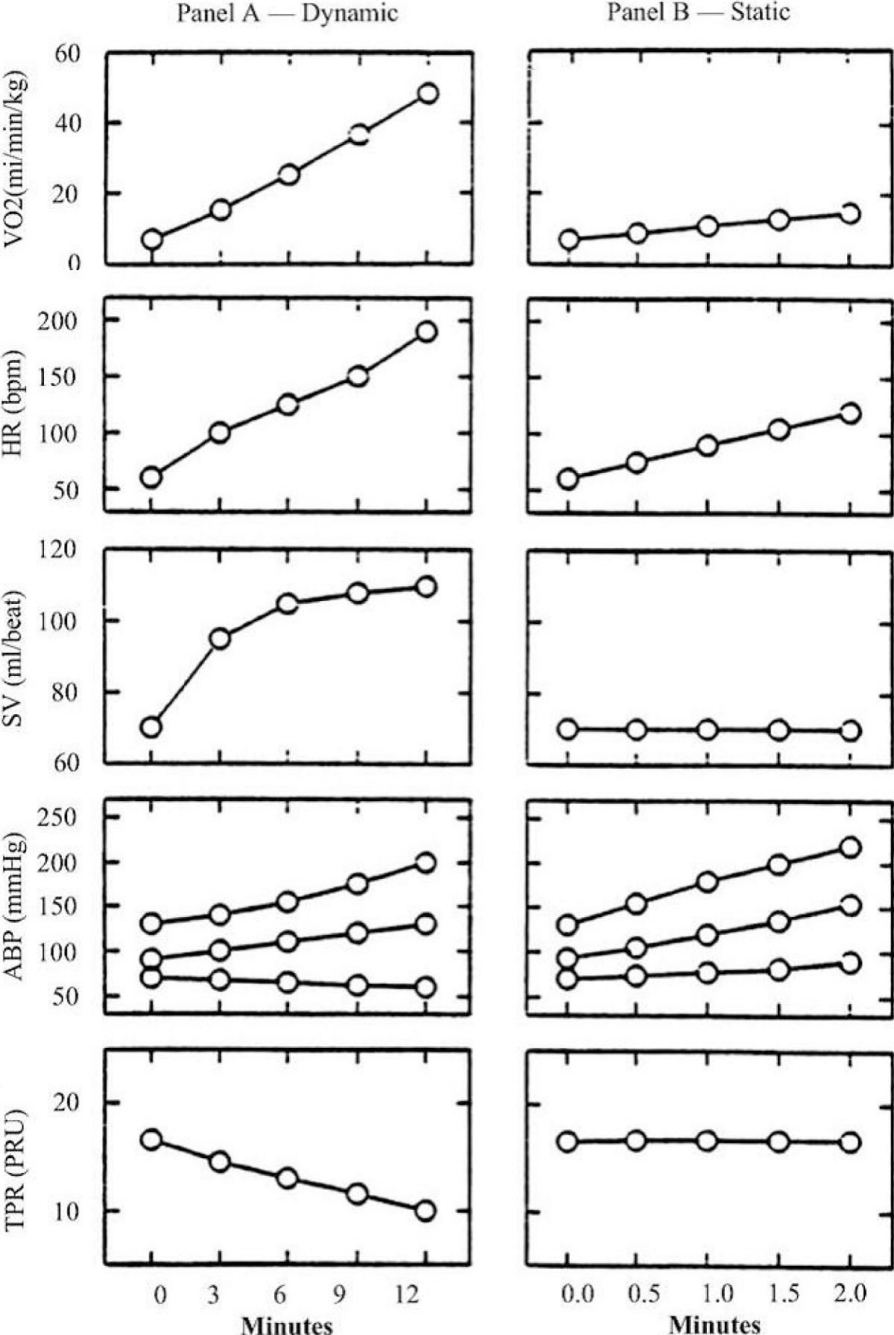
**Cardiovascular response to physical exercise** Legend: **(A)** Response to dynamic exercise gradually to the increase in workload to the maximum oxygen consumption; **(B)** Response to static exercise (handgrip at 30% of maximum voluntary contraction); VO2 (ml/min/kg); HR (bpm) Heart rate; SV (ml/beat) Stroke volume; ABP (mmHg): systolic, diastolic and mean arterial pressure; TPR (PRU) peripheral vascular resistances (expressed in peripheral resistance Unit)

The first is a type of exercise that involves an increase in cardiac output associated with rapid increases in heart rate and systolic BP (with a minimum decrease in diastolic BP) but with a significant decrease in peripheral vascular resistance. The isometric exercise, or static, involves a sharp increase in both systolic and diastolic blood pressure, a modest increase in heart rate with a range stable or slightly reduced but no decrease in peripheral vascular resistance. In Figure 1 are shown the modifications and interactions between the various parameters [1].

From these premises it emerges therefore the need to start young people suffering for arterial hypertension to play a type of sport that is suitable for the individual cardiovascular status.

In Italy, compared to the US, to practice a sport activity is required medical certification that involves a civil and criminal liability on the part of the physician certification and which is regulated by specific decrees, in particular the latest amendments to the Balduzzi Decree [2]. In the case of healthy children, or apparently healthy, it is scheduled to perform at least once a 12-lead ECG at rest and blood pressure measurement (as specifically stated on the label of certification then signed at the bottom by a doctor). In the case of children and adolescents with arterial hypertension, both for primary and secondary hypertension, you need to pay more attention and care, both in relation to cardiovascular stresses that this entails as previously exposed, both in relation to the importance of implementing the changes in lifestyle who are always the first step, no drugs, which will contribute to the maintenance of blood pressure in the normal range. For what concerns instead the practice of competitive sports activities, please refer to specialists in Sports Medicine, which is reserved for the certification.
